# Antioxidants and Dementia Risk: Consideration through a Cerebrovascular Perspective

**DOI:** 10.3390/nu8120828

**Published:** 2016-12-20

**Authors:** Virginie Lam, Mark Hackett, Ryusuke Takechi

**Affiliations:** 1 Curtin Health Innovation Research Institute, Curtin University, Perth WA 6845, Australia; Virginie.Lam@curtin.edu.au (V.L.); Mark.J.Hackett@curtin.edu.au (M.H.); 2 School of Public Health, Faculty of Health Sciences, Curtin University, Perth WA 6845, Australia; 3 Department of Chemistry, Faculty of Science and Engineering, Curtin University, Perth WA 6845, Australia

**Keywords:** antioxidants, blood-brain barrier, cognitive impairment, dementia

## Abstract

A number of natural and chemical compounds that exert anti-oxidative properties are demonstrated to be beneficial for brain and cognitive function, and some are reported to reduce the risk of dementia. However, the detailed mechanisms by which those anti-oxidative compounds show positive effects on cognition and dementia are still unclear. An emerging body of evidence suggests that the integrity of the cerebrovascular blood-brain barrier (BBB) is centrally involved in the onset and progression of cognitive impairment and dementia. While recent studies revealed that some anti-oxidative agents appear to be protective against the disruption of BBB integrity and structure, few studies considered the neuroprotective effects of antioxidants in the context of cerebrovascular integrity. Therefore, in this review, we examine the mechanistic insights of antioxidants as a pleiotropic agent for cognitive impairment and dementia through a cerebrovascular axis by primarily focusing on the current available data from physiological studies. Conclusively, there is a compelling body of evidence that suggest antioxidants may prevent cognitive decline and dementia by protecting the integrity and function of BBB and, indeed, further studies are needed to directly examine these effects in addition to underlying molecular mechanisms.

## 1. Introduction

As a consequence of rapidly aging populations, particularly in developed nations, dementia has become a major health and medical issue imposing an extraordinary economic burden. As reported by the World Health Organization and Alzheimer’s disease International, the global cost of dementia-related healthcare was estimated to be $604 billion in 2010, which was equal to 1% of world gross domestic product, indicating a significant socioeconomic impact [1]. Studies also predict that this cost will greatly increase and is expected to double in the next 10–15 years. Indeed, the latest estimated global cost of dementia in 2015 based on a meta-analysis was $818 billion, an increase of 35% since 2010 [2]. Astoundingly, the estimated prevalence of dementia has increased from 35.6 million in 2010 to 46.8 million in 2015, an increase of 34% [2]. Clearly, there is an urgent necessity to establish effective therapeutic strategies to delay or prevent the onset and progression of this disorder.

Major subtypes of dementia are Alzheimer’s disease (AD), vascular dementia, Lewy body dementia, and frontotemporal dementia, which accounts for approximately 43%, 15%, 5% and 1% of all dementia cases, respectively [3]. Although the pathology and pathogenesis of these disorders remain largely unclear, it is increasingly recognized that the integrity of cerebrovasculature is critical to the maintenance of healthy brain function and integrity [4]. The human brain ordinarily receives 20% of cardiac output despite its small volume (2% against total body mass), and the surface area of cerebrovascular network available for molecular exchange between the brain and blood is approximately 20 m^2^ [5]. Dysfunctional cerebrovascular integrity allows blood-to-brain extravasation of potentially neuroactive molecules, which thereafter trigger a neuroinflammatory cascade and subsequently, activation of neuronal apoptosis pathways, conditions which lead to neurodegeneration and if persisting, cognitive decline. Thus, it is highly plausible that subtle changes in cerebrovascular permeability can have substantial impacts on the brain and neurocognitive function.

In recent clinical and animal model studies, agents with anti-oxidative properties are reported to exert therapeutic effects on cognitive impairment and dementia [6,7,8]. However, whilst the majority of these studies demonstrated the beneficial effects of antioxidants on cognitive function via direct neuroprotective actions within the brain, no studies have implicated the efficacy of antioxidant therapy through the cerebrovascular axis. Therefore, this review summarizes the current available data from both animal and human studies to potentiate the role of antioxidants in the prevention of dementia and cognitive decline via mechanisms mediated through the cerebrovascular axis. Moreover, considerations for future studies examining the antioxidant effects on the cerebrovasculature are discussed.

## 2. Cerebrovascular Integrity in Neurodegeneration, Cognitive Decline and Dementia

The brain is a vital organ, yet extremely vulnerable to various endogenous and exogenous insults such as viral and bacterial pathogens, inflammatory cells, pro-inflammatory cytokines, reactive oxygen species (ROS), and macronutrients [5]. Therefore, in a healthy, non-pathological state, this organ is protected from the peripheral circulation by a structurally unique neurovascular unit, which constitutes the blood-brain barrier (BBB). The main feature of the BBB is a monolayer of endothelial cells that are tightly opposed to one another, forming a physical barrier between the brain and blood. The cells are fused each other by tight junctional and adheren junctional complexes, which consist of integral membrane tight junction proteins including occludin and claudin, anchored by cytoplasmic zonula occludens (ZO). This impermeable layer of endothelial cells is structurally supported by several layers of underlying basement membranes as well as pericytes and astrocytic endfeet. A healthy, functioning BBB strictly regulates molecular trafficking between the brain and blood, allowing only highly specific transcellular transport of particular molecules that are essential to the brain, such as glucose and oxygen. However, under certain pathological stress conditions, the integrity of this highly selective barrier system can be transiently or chronically compromised depending on the nature of the insults. The factors that can deteriorate BBB integrity and increase its permeability include inflammation, oxidative stress, hypertension, stroke, HIV, lipids, smoking, alcohol intake, mental stress, and lowered cerebral blood flow, although the underlying molecular mechanisms are not fully understood [4,9,10].

### 2.1. Dysfunction of the Cerebrovascular Blood-Brain Barrier

A dysfunctional BBB may occur via (i) impaired transcellular transport, mainly due to dysregulation of endothelial receptors and intracellular transporters; (ii) impaired paracellular unspecific extravasation of molecules due to a loss of tight junction complex; and/or (iii) loss of endothelial cells per se due to apoptosis or traumatic injury. Persistent disturbances of BBB allow substantial cerebral extravasation of blood-borne potentially neurotoxic molecules, which thereby promote the synthesis and release of pro-inflammatory cytokines resulting in the activation of microglial phagocytes and production of ROS [11]. Chronically heightened inflammatory and oxidative stress in the brain results in the production of toxic products that compromise cell function, alter cellular phenotypes, damage DNA, and eventually lead to neuroinflammation and neurodegeneration. The latter is increasingly suggested by a number of studies to be profoundly associated with the pathogenesis and pathology of cognitive decline and dementia including AD and vascular dementia [5]. Indeed, neuroinflammation, microgliosis and mitochondrial dysfunction are commonly observed in the brain of subjects with AD and vascular dementia [12,13,14].

### 2.2. Blood-Brain Barrier Dysfunction in Cognitive Deficits and Dementia

Despite decades of attempts in Alzheimer’s research, anti-amyloid strategies have failed to consistently deliver positive results in the prevention and treatment of AD [7,15]. In fact, some neuropsychological and neuropathological characteristics of AD cannot be fully explained by the amyloid hypothesis alone. For example, some studies report that the severity of amyloidosis does not consistently correlate with the severity of AD, and the biosynthesis of β-amyloid in sporadic AD subjects is comparable to otherwise healthy individuals [9,16,17].

Emerging evidence substantiates the involvement of cerebrovascular dysfunction in the pathogenesis of AD and other dementias. In vivo and in vitro studies demonstrated that the transport of β-amyloid across the BBB is mediated by receptor for advanced glycation endproducts [18,19]. Thus, the dysregulation of such pathways is suggested to result in reduced β-amyloid clearance from the brain, which may consequently induce amyloid plaque formation. The vascular risk factors have been increasingly associated with heightened risk of dementia and AD, whereby a substantial body of work has reported exaggerated BBB permeability in the brains of individuals with cognitive impairment or dementia [4]. Post-mortem histological analyses revealed an accumulation of blood-borne proteins including albumin, immunoglobulins, and fibrinogen within the parenchyme of hippocampal formation and cortical regions of subjects with AD [20]. In addition, other post-mortem studies reported degeneration of BBB pericytes and reduced endothelial tight junction protein expression in human AD brain [21]. Further supportive evidence of BBB breakdown in individuals with AD and mild cognitive impairment is provided by studies showing increased concentration of albumin in cerebrospinal fluid relative to blood [22,23]. Moreover, signs of compromised BBB integrity are commonly reported in animal models of AD, leading to loss of endothelial tight junction complex and cerebral extravasation and accumulation of plasma derived macromolecules [9,24]. These data consistently implicate a strong association between dysfunctional BBB and cognitive decline and dementia.

There has been rigorous debate on whether the breakdown of BBB is a primary causative factor of neurodegeneration and dementia or whether it occurs as a downstream response [25]. An increasing number of studies to date report that BBB disruption precedes neurodegeneration and cognitive decline in clinical AD subjects, and in AD animal models, the appearance of hallmark pathophysiological features such as amyloid plaques, strongly suggesting a causal link rather than a consequential one [4]. Recent advances in neuroimaging and neurovascular imaging technologies using state-of-art dynamic contrast-enhanced MRI now allow non-invasive evaluation of regional BBB permeability in vivo with high sensitivity [26]. In 2016, an intriguing study from Maastricht University Medical Center elegantly demonstrated the substantially increased permeability of BBB at a very early stage of AD [27]. The study also revealed that the extent of BBB leakage positively associates with the severity of cognitive decline measured by Mini-Mental State Examination. In addition to this recent report, BBB breakdown was demonstrated to be evident in a murine model of AD Tg2576 mice as early as 4 months of age, which was 10 months before the distinct formation of amyloid plaques at 14 months [28]. Moreover, a recent study in our laboratory demonstrated that substantial disruption of BBB and neuroinflammation was observed prior to neurodegenerative changes and cognitive decline in a dietary-induced mouse model of cognitive decline (unpublished observations by Takechi et al.). Collectively, compelling evidence from experimental and clinical studies strongly indicate a causal association between compromised BBB integrity and the onset and progression of cognitive dysfunction and dementia.

## 3. Involvement of Oxidative Stress during Breakdown of the Blood-Brain Barrier

Oxidative stress is a state that arises from an inappropriate redox balance where the production of ROS exceeds the conversion/neutralization of ROS into less toxic derivatives by antioxidants such as glutathione and superoxide dismutase (SOD). ROS may be generated by several pathways, such as conversion of oxygen into superoxide by enzymatic activities of oxidase or altered metal homeostasis (Mn, Fe, Cu) and ensuing metal catalyzed free radical production through classic Fenton chemical pathways. Superoxide is frequently converted into hydrogen peroxide, the most bioactive and stable form of ROS, however, superoxide may also react with nitric oxide generating additional free radicals. In addition, hydrogen peroxides can also be generated directly by some enzymes such as NADPH oxidase. Therefore, oxidative stress can occur by an overproduction of ROS by enzymes including NADPH oxidase, and/or from lowered antioxidant levels or activity including SOD. Studies report that elevated oxidative stress may have direct and indirect effects on the integrity of BBB [29].

**Figure 1:**
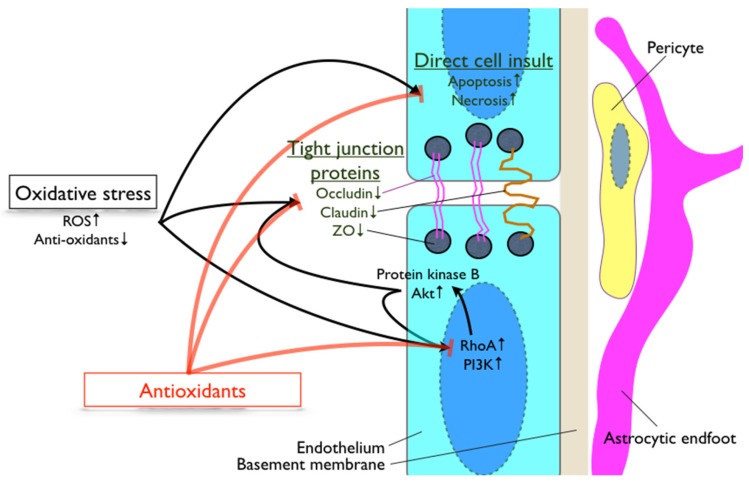
Involvement of oxidative stress in blood-brain barrier regulation. The diagram summarizes the oxidative stress pathways that are involved in the regulation of blood-brain barrier integrity. PI3K: phosphoinositide 3-kinase; RhoA: ras homolog gene family member A; ROS: reactive oxygen species; ZO: zonnula occluden.

An involvement of oxidative stress in the regulatory mechanisms of BBB integrity is supported by a number of studies [29,30,31,32] (Figure 1). A study using SOD deficient mice demonstrated a substantial breakdown of BBB allowing an extravasation of large blood-borne molecules into the brain [33]. Lochhead et al. demonstrated that oxidative stress induced through a process of hypoxia and reoxygenation in rats altered occludin structure and localization in cerebrovascular endothelial cells [30]. Increased oxidative stress disrupted BBB by attenuating the expression of tight junction proteins, ZO-1, occluding and claudin in diabetic mode rats [34]. In vitro studies confirmed ROS-mediated modulation of BBB permeability occurs in a time- and concentration-dependent manner [35,36]. Furthermore, Schreibelt et al. revealed in an in vitro model of BBB, ROS-induced BBB disruption occurred through a loss of tight junction claudin-5 and occludin was mediated by upregulated RhoA and PI3 kinase [37]. The study also for the first time reported the involvement of protein kinase B/Akt in ROS-induced alteration of BBB tight junction expression and localization. Moreover, a study using bovine brain microvascular endothelial cells indicated that increased superoxide induced F-actin stress fiber formation through a Rho-dependent pathway [37]. ROS is also documented to increase the expression of chemokine receptors, resulting in signaling flux and phosphorylation of myosin light chain, which thereafter modulates actin structure [38]. The latter findings are consistent with the cytoskeletal reorganization of BBB promoting the loss of BBB integrity.

Overall, the current literature implicates the substantial involvement of oxidative stress in BBB dysfunction (Figure 1), and hence implies the pleiotropic effects of antioxidants in the protection of BBB function and structure.

## 4. Antioxidants, Cognitive Decline and Blood-Brain Barrier

Studies report that oxidative stress is positively associated with impaired cognitive function [39,40]. Consistently, multiple lines of evidence demonstrate beneficial effects of antioxidants on cognition and dementia [8]. As a wealth of recent evidence suggests that BBB dysfunction is associated with the pathogenesis and pathology of neurodegeneration and the associated-cognitive deficits, the preservation of BBB structure and function may offer an innovative and robust therapeutic opportunity for cerebrovascular disorders. Whilst a number of studies have implicated antioxidant therapy as a therapeutic option against cognitive decline, few studies have considered the positive effects of antioxidants on cognition and dementia through a neurovascular axis. Therefore, in this chapter, we will summarize the available literature that report the effects of antioxidants on BBB integrity and cognition, and consider its use in dementia therapy through a cerebrovascular perspective (refer to Table 1 for summary).

### 4.1. Anti-Oxidative Vitamins

A number of studies report that diet and nutrients have a strong association to the risk of dementia including AD [41,42]. Vitamin A, C and E are known as potent anti-oxidants. In concert with the substantial involvement of oxidative stress pathways in the pathophysiology of AD, studies found that plasma and cerebrospinal fluid concentrations of Vitamins A and C were significantly lower in patients with AD despite comparable dietary intake to healthy control subjects [43,44]. In a Rotterdam study involving 5395 participants, high intake of dietary Vitamin C and Vitamin E was significantly associated with lowered risk of AD and dementia [45,46]. A prospective population study with 4740 participants revealed that the use of Vitamin C and E supplements for more than 3 years significantly reduced the risk of AD [47]. Furthermore, a randomized control trial by Li et al. showed co-supplementation of Vitamin E and C with β-carotene (another well-established dietary antioxidant) markedly improved cognitive function in otherwise healthy elderly individuals [48]. Consistent with these clinical findings, an animal study reported that acute treatment with Vitamin C attenuates the spatial learning and memory in APP/PSEN1 transgenic AD model mice and also in aged none-AD wild-type mice [49]. However, in the same study, supplementation of Vitamin C did not alter cerebral redox state, inflammation and amyloidosis, indicating that the anti-dementia effects of vitamins may not only be attributed to its anti-oxidative properties. Indeed, Vitamin C has been reported to prevent the oligomerization of Aβ [50]. An oral administration of Vitamin C is also reported to significantly suppress cerebral oxidative damage in cerebral ischemia-reperfusion [51]. In addition, in vivo and in vitro studies report that Vitamin A prevents neurodegeneration, and inhibits the formation of Aβ fibrils, decreasing its aggregation and oligomerization [44,52].

It has been suggested that anti-oxidative vitamins may prevent cognitive decline also by protecting BBB integrity. However, only a few studies have directly investigated the effects of anti-oxidative vitamins on dementia through cerebrovascular axis. A study by Kook et al. recently reported that high dose supplementation of Vitamin C reduced amyloidosis in the cortex and hippocampus of AD mice (5XFAD) via attenuation of BBB disruption and mitochondrial alteration [53]. Vitamin C is reported to prevent the disruption of BBB induced by compression of primary somatosensory cortex by upregulating the expression of tight junction proteins, occludin and claudin-5 [54]. In a model of stroke with substantial BBB disruption, a single injection of Vitamin C significantly reduced BBB permeability [55]. Similarly, in a mouse model of cerebral ischemia, Vitamin C prevented BBB dysfunction by protecting tight junction claudin-5 and attenuated edema and neuronal loss [56]. Moreover, an in vitro study provides supporting data that Vitamin C reversed hyperglycemia-mediated BBB disruption [57]. Similar to Vitamin C, Vitamin E is also reported to prevent BBB breakdown induced by oxidative stress by attenuation of endothelial oxidative stress and via increasing the expression of tight junction proteins in vivo [58]. In rats with convulsion under hyperthermic conditions, Vitamin E showed beneficial effects on modulation of BBB permeability [59]. Consistent with the latter, ingestion of Vitamin E deficient diet in wild-type rats resulted in increased BBB permeability and heightened oxidative stress [60].

Although the underlying mechanisms by which anti-oxidative vitamins protect BBB integrity are largely unknown, these studies collectively suggest that these vitamins may ameliorate neurodegenerative changes and cognitive decline by preserving the function and structure of BBB. More direct experimental and clinical evidence would provide further strength to such hypotheses.

### 4.2. Other Natural Antioxidants

Melatonin is a neurohormone found in animals, plants and bacteria, reported to exhibit strong anti-oxidative properties through protecting the mitochondrial membrane potential and thereby suppressing the production of superoxides [91,92]. Melatonin is approved by the FDA as a dietary supplement and readily available over the counter, commonly prescribed for treatment of insomnia. Studies report that plasma melatonin levels are significantly decreased in subjects with AD, showing a negative association with cognitive function, and a significant reduction is found even in healthy elderly [93,94,95]. In a rat model of sporadic AD, melatonin was demonstrated to prevent memory loss by attenuating amyloidosis and neurodegeneration [96]. Furthermore, in aged 22-month old wild-type mice with significantly impaired spatial cognition, melatonin improved cognitive performance by reducing cerebral amyloid burden through altered protein-cleaving secretase expression [97]. In addition, some studies showed that melatonin promotes hippocampal neuroplasticity and stimulates the proliferation and differentiation of neural stem cells in vitro and in vivo [98,99,100]. A number of studies have also demonstrated protective effects of melatonin on the BBB. A study by Vornicescu et al. demonstrated that melatonin administration prevents oxidative stress-induced breakdown of BBB and improves cognitive function [61]. In this study, a significant reduction in neuronal death and neuroinflammation was also observed in melatonin treated rats. In vitro studies further investigated mechanistic insights into the BBB protective effects of melatonin. A study using rat brain microvascular endothelial cells revealed that BBB disruption induced by inflammation was completely prevented by melatonin treatment via restoration of the expression of BBB tight junction ZO-1 and inhibiting matrix metalloproteinase-9 [62]. Similarly, in a bEnd.3 cell line model of oxidative stress-induced BBB dysfunction, treatment with melatonin prevented cell death and degradation of tight junction proteins by activating Akt and suppressing phosphorylation of JNK [63]. Overall, the data presented strongly suggests that melatonin may exert neuroprotective effects partially, if not entirely, through the protection of BBB from oxidative and inflammatory stressors.

α-Lipoic acid (ALA) is known to chelate transition metals and ROS, thus inhibiting hydroxyl radical formation. A clinical trial involving 43 AD patients over an observation period of 2 years demonstrated that subjects receiving ALA showed constant scores in two neuropsychological tests, while the untreated subjects showed significant decline in cognitive function [101]. Consistent with the latter, in a rat model of vascular dementia, ALA significantly restored cognitive functioning concomitant with markedly reduced ROS and malondialdehyde production and increased reduced glutathione levels in the hippocampal formation, the domain crucial to learning and memory [102]. Increased acetylcholine and choline acetyltransferase levels as well as decreased acetylcholinesterase activity were also observed in the hippocampus of ALA-treated rats. By using a murine model of accelerated ageing, senescence accelerated mouse prone-8, ALA was demonstrated to improve memory and supress oxidative stress during normal ageing [103]. However, few studies to date have directly considered BBB permeability in context of the neuroprotective properties of ALA. Takechi et al. reported in a dietary induced mouse model of BBB dysfunction, ALA preserved the integrity of BBB by inhibiting oxidative stress, which resulted in the prevention of blood-to-brain protein extravasation and significant attenuation of neuroinflammation [64]. Similarly, ALA was reported to ameliorate BBB disruption, neuroinflammation and neurological motor deficits in a rat model of ischemic stroke [65]. Although these studies strongly suggest that ALA may prevent cognitive decline through the protection of BBB, further studies are needed to directly investigate such association.

Another emerging potentially neuroprotective antioxidant is garlic. An extract of garlic contains a mixture of biologically active compounds such as S-allylcysteine (SAC) and S-allylmercaptocysteine, which are known to exert strong anti-oxidative effects. These compounds have repeatedly been shown to provide antioxidant action by scavenging ROS, enhancing SOD, catalase glutathione peroxidase, and increasing glutathione. A number of studies demonstrated the neuroprotective effects of garlic [104]. Although clinical evidence is limited, Chauhan et al. elegantly demonstrated the beneficial effects of garlic extracts in AD models [105]. In a study using Alzheimer’s Tg2576 mice supplemented with either aged garlic extract (AGE), SAC, or di-allyl-disulphide (DADS) for 4 months, mice treated with AGE as well as mice treated with SAC or DADS showed substantially lower cerebral amyloidosis compared to untreated mice [106]. Similarly, AGE, SAD and DADS significantly attenuated neuroinflammation and tau protein. The extent of the neuroprotective effects reported were ranked from AGE > SAC > DADS. Furthermore, a study using TgCRND8 AD model mice revealed that the provision of AGE improved hippocampal-dependent cognition and memory [107]. Additionally, in a study by Takechi et al., the cerebrovascular-protective effects of AGE via its anti-oxidative properties were demonstrated in a mouse model of BBB dysfunction, which coincided with significant attenuation of neuroinflammation [64]. In vitro and in vivo studies confirmed that the pleiotropic effects of garlic extracts may be attributed to its anti-oxidative action on neurons, including sympathetic neurons, preventing ROS-mediated oxidative insults [108]. The studies also revealed that SAC was the most potent neuroprotective compound in AGE [108]. The data collectively suggests that AGE, or its anti-oxidative components, may exert neuroprotective effects through various pathways including direct anti-oxidative protection of neuronal cells as well as the preservation of BBB integrity.

There are other natural antioxidants that are recently reported to exhibit potent BBB protective effects. These include apocynin [66,67,68], baicalein [69], caffeine [70,71,109], curcumin [72,73,74], niacin [64], nicotine [64], pinocembrin [75,76,110], resveratrol [77,78,79,80,81,111], polyphenols [112], and tanshinone IIA [82,83,84]. However, the number of studies investigating the effects of these agents on neurodegeneration and cognitive performance is limited. Further investigations to support the use of these agents for dementia prevention by focussing on the neurovascular integrity need to be conducted.

### 4.3. Lipid-Lowering Drugs with Anti-Oxidative Properties

Many clinical drugs for treatment of cardiovascular disease exert antioxidant effects. Statins, 3-hydroxy-3-methylglutaryl coenzyme A reductase inhibitors, are reported to show anti-oxidative properties by inhibiting the increase of 8-isoprostane and suppressing the activity of nitric oxide synthase [113,114]. While its effects on dementia are still controversial [115,116,117], case control and retrospective population studies indicate that statins significantly reduce the risk of dementia and cognitive impairment [118]. A 7-year follow-up study, the Rotterdam Study, revealed that statin users have a 43% lower incidence of AD [119]. A number of putative underlying mechanisms for statin’s neuroprotective effects are suggested [120]. Studies in humans and animal models showed that statins attenuate cerebral amyloidosis in AD [121,122]. Other studies showed that cerebral oxidative stress and the production of pro-inflammatory cytokines were reduced by atorvastatin in animal models of AD [123,124]. An emerging body of recent studies also suggests statins’ therapeutic potential for AD via protection of the neurovascular unit [125]. In a transgenic mouse model of AD, atorvastatin and pitavastatin improved memory and reduced amyloidosis and neuroinflammation by modulating the permeability of BBB. Similarly, simvastatin was shown to protect BBB integrity and attenuate neuropathophysiology and oxidative stress in APP transgenic AD mice [126]. In line with the latter, an in vitro study using human cerebral microvascular endothelial cells showed that simvastatin and lovastatin attenuated Aβ-induced BBB dysfunction [85]. Moreover, in a mouse model of dietary- induced BBB dysfunction, we demonstrated that atorvastatin and pravastatin reversed the disruption of BBB integrity [86]. Interestingly, all studies indicated here reported the BBB protective effects of statins independent of their lipid-lowering effects but rather occurred concomitant with attenuated oxidative stress, suggesting its cerebrovascular protective action through anti-oxidative pathways.

Probucol is a conventional cholesterol-lowering drug that possesses potent anti-oxidative properties, which attribute to attenuation of ROS by inhibiting the expression of Nox2 [127], and by increasing glutathione peroxidase activity [128]. A limited number of clinical trials report that probucol stabilizes cognitive function in AD [129]. Consistent with this notion, a study reported in a mouse model of cognitive and hippocampal synaptic impairment induced by an intracerebroventricular injection of aggregated Aβ_1-40_, probucol prevented hippocampal lipid peroxidation and attenuated loss of hippocampal-dependent learning and memory [130]. Similarly, in a mouse model of streptozotocin-induced cognitive impairment, probucol improved cognitive function by attenuating hippocampal oxidative stress [131]. Consistent with the hypothesis, studies in our laboratory reported that probucol prevented the disruption of BBB and neuroinflammation induced by an ingestion of a high-fat diet in mice [87,88]. These studies also revealed that the BBB protective effects of probucol was through its anti-oxidative effects by attenuating cerebral oxidative stress and neurovascular inflammation. A study by Russell et al. further supports this data by showing improved vascular endothelial function in a model of atherosclerosis by administration of probucol, independent of its cholesterol-lowering effects [132].

Fenofibrate is another lipid-lowering drug reported to lower cholesterol and triglycerides and increase HDL-cholesterol [133]. Besides its anti-atherosclerotic effects, fenofibrate is reported to exert anti-oxidative effects by reducing lipid peroxidation and increasing antioxidants including glutathione, in a rat model of Parkinson’s disease [134]. Interestingly, in the same study by Uppalapati et al., fenofibrate attenuated neurodegeneration and improved cognitive function in a dose-dependent manner [134]. Consistently, in a brain irradiation-induced mouse model of cognitive impairment, fenofibrate significantly improved cognitive performance [135]. However, the clinical evidence of fenofibrate on cognitive function and dementia is limited [136]. In a murine model of BBB dysfunction induced by HIV Tat protein, Huang et al. demonstrated that fenofibrate attenuated BBB permeability, neuroinflammation and neurodegeneration [89]. Moreover, an in vitro study using mouse cerebral capillary endothelial cells showed that fenofibric acid (an active metabolite of fenofibrate) protected BBB integrity from oxygen-glucose deprivation-induced BBB hyper-permeability [90]. However, no studies to date have considered the neuroprotective effects of fenofibrate through modulation of BBB-specific pathways.

These studies suggest that the lipid-lowering pharmacological agents with anti-oxidative properties exert positive effects on neurodegeneration and cognitive deficits, at least in part, through protective mechanisms via the BBB. Further studies to identify more detailed molecular and cellular mechanisms to strengthen this evidence are required.

### 4.4. Other Pharmacological Agents with Anti-Oxidative Effects

There are several other pharmacological agents with anti-oxidative properties exerting vascular protective effects, and some are demonstrated to be beneficial for BBB. Ibuprofen is a widely utilized nonsteroidal anti-inflammatory drug (NSAID) reported to non-selectively attenuate the activity of cyclooxygenase and is reported to exert substantial oxidative effects [137]. A number of epidemiological and clinical studies reported that the use of ibuprofen and other NSAIDs delayed the onset and progression of AD [138,139]. Although the mechanisms by which NSAIDs reduce the risk of AD are yet to be elucidated, both in vivo and in vitro studies showed that NSAIDs including ibuprofen, flurbiprofen, indomethacin and sulindac, significantly attenuated the cerebral production and accumulation of Aβ [140] and suppressed neuroinflammation [141]. Combination therapy of ibuprofen with lipoic acid was also reported to show neuroprotective effects in a rat model of AD [142]. A recent study revealed significant alterations in protein and phosphoprotein expression including heat shock protein 8, dihydropyrimidinase-related protein 2 and γ-enolase, within the hippocampal formation [143]. In addition, a recent study reported that ibuprofen prevents the decrease of N-acetylaspartate and atrophy of hippocampal formation in a transgenic mouse model of AD via MRI neuroimaging and spectroscopy analysis [144]. However, none of these studies considered the effect of ibuprofen on cerebrovascular integrity. To our best knowledge, the only study demonstrating BBB modulating effects of ibuprofen is that by Pallebage-Gamarallage et al. [86]. The study reported novel findings in which ibuprofen treatment for 1 or 3 months can restore the compromised BBB integrity in a high fat-induced BBB dysfunction mouse model. Clearly, further investigation is necessary to elucidate the BBB protective role of ibuprofen in cognition and dementia.

## 5. Conclusions

Previous studies suggest that natural and chemical compounds with anti-oxidative properties have beneficial effects on neuroprotection and cognitive performance, although the underlying mechanisms are largely unknown. As summarized in the current review, clinical evidence suggests the therapeutic effects of antioxidants on dementia and cognitive impairment and findings from animal and in vitro studies support this evidence. Furthermore, a substantial body of evidence from animal and in vitro studies suggests that such neuroprotective effects of antioxidants may be entirely or partially attributed to the BBB protective effects of antioxidants. However, the number of studies directly considering the therapeutic effects of antioxidants on cognitive impairment through the axis of cerebrovascular BBB integrity remain limited. Further investigations should focus on simultaneously testing the neuroprotective effects as well as the BBB protective effects of antioxidants. Furthermore, the elucidation of such mechanisms may lead to the development and search for new agents to specifically target the protection of BBB, conferring neuroprotection.

## Figures and Tables

**Table 1:** Literature summary.

Antioxidants	Condition	Study Type/Model	Neuronal Measure(s)	Blood-Brain Barrier Measure(s)	Others	Reference No.
Vitamin C&E	cerebral ischemia	mouse model	↓ neuronal loss	↑ claudin-5		[54]
	AD	mouse model (5XFAD)	↓ amyloid plaques	↓ BBB dysfunction		[51]
	BBB disruption	mouse model	N/A	↑ occludin, claudin-5		[52]
	stroke	rat model	N/A	↓ BBB dysfunction		[53]
	hyperglycemia	HBMEC	N/A	↓ BBB dysfunction		[55]
Vitamin E	Phospholipid transfer protein deficient	mouse model	N/A	↑ occludin, claudin-5, ZO-1		[56]
	healthy	rats under hyperthermic convulsion	N/A	↓ BBB dysfunction		[57]
	healthy	rats with vitamin E deficient diet	N/A	↓ BBB dysfunction		[58]
Melatonin	hypobaric hypoxia	rats	↑ cognitive function; ↓ neuronal loss, neuroinflammation	↓ BBB dysfunction		[61]
	inflammation	rat brain microvascular endothelial cells	N/A	↑ ZO-1		[62]
	oxidative stress	bEnd.3 cells	N/A	↑ claudin-5; ↓ cell death		[63]
α-Lipoic acid	high-fat diet	mouse model	↓ neuroinflammation	↓ BBB dysfunction		[64]
	ischemic stroke	rat model	↓ neurological deficit, neuroinflammation	↓ BBB dysfunction		[65]
Aged garlic extract	high-fat diet	mouse model	↓ neuroinflammation	↓ BBB dysfunction		[64]
Apocynin	BBB disruption	rat perfusion model	N/A	↓ BBB dysfunction	improved vascular tone	[66]
	BBB disruption	HBMEC	N/A	↓ BBB dysfunction	↑ AMPK activation	[67]
	BBB disruption	HBMEC	N/A	↑ occludin, claudin-5	↑ AMPK activation	[68]
Baicalein	intracerebral hemorrhage	rat model	↓ neurological deficit	↑ ZO-1		[69]
Caffein	Parkinson’s disease	mouse model	N/A	↑ occludin, ZO-1		[70]
	AD	rabbit model	↓ neuroinflammation	↑ occludin, ZO-1		[71]
Curcumin	subarachnoid hemorrhage	rat model	↓ neurological deficit, neuroinflammation	↓ BBB dysfunction		[72]
	cerebral ischemia	rat model	↓ neurological deficit	↓ BBB dysfunction	↓ infarct volume	[73]
	N/A	BMEC	N/A	↓ platelet recruitment		[74]
Pinocembrin	cerebral ischemia	rat model	↓ neurological deficit	↓ BBB dysfunction	↓ brain edema	[75]
	cerebral ischemia	rat model	↓ neuroinflammation	↑ occludin, ZO-1		[76]
Resveratrol	autoimmune encephalomyelitis	mouse model	↓ neuroinflammation, oxidative stress	↑ occludin, ZO-1, claudin-5; ↓ ICAM-1, VCAM-1		[77]
	BBB disruption	HBMEC	↓ oxidative stress	↓ BBB dysfunction		[78]
	AD	rat model	↓ neuroinflammation, β-amyloid	↑ claudin-5		[79]
	cerebral ischemia	rat model	↓ neuronal loss	↓ BBB dysfunction	↓ brain edema	[80]
	high-fat diet	mouse model	↓ neuronal loss	↑ occludin, ZO-1		[81]
Tanshinone IIA	autoimmune encephalomyelititis	mouse model	↓ neuroinflammation	↑ occludin, claudin-5, ZO-1		[82]
	hypoxia	HBMEC	N/A	↑ ZO-1		[83]
	cerebral ischemia	rat model	N/A	↑ occludin, ZO-1; ↓ ICAM-1	↓ brain edema	[84]
Statin	AD	in vitro BBB model	N/A	↓ BBB dysfunction		[85]
	high-fat diet	mouse model	N/A	↓ BBB dysfunction		[86]
Probucol	high-fat diet	mouse model	↓ neuroinflammation	↓ BBB dysfunction		[87,88]
Fenofibrate	BBB disruption	mouse model	↓ neurodegeneration, neuroinflammation	↓ BBB dysfunction		[89]
	BBB disruption	BMEC	N/A	↓ BBB dysfunction		[90]
Ibuprofen	high-fat diet	mouse model	N/A	↓ BBB dysfunction		[86]
